# Identifying the ‘red flags’ for unhealthy weight control among adolescents: Findings from an item response theory analysis of a national survey

**DOI:** 10.1186/1479-5868-9-99

**Published:** 2012-08-21

**Authors:** Jennifer Utter, Simon Denny, Elizabeth Robinson, Shanthi Ameratunga, Sue Crengle

**Affiliations:** 1 School of Population Health, University of Auckland, Private Bag 92019, Auckland, New Zealand

**Keywords:** Disordered eating, Weight loss, Adolescents, Item response theory

## Abstract

**Background:**

Weight control behaviors are common among young people and are associated with poor health outcomes. Yet clinicians rarely ask young people about their weight control; this may be due to uncertainty about which questions to ask, specifically around whether certain weight loss strategies are healthier or unhealthy or about what weight loss behaviors are more likely to lead to adverse outcomes. Thus, the aims of the current study are: to confirm, using item response theory analysis, that the underlying latent constructs of healthy and unhealthy weight control exist; to determine the ‘red flag’ weight loss behaviors that may discriminate unhealthy from healthy weight loss; to determine the relationships between healthy and unhealthy weight loss and mental health; and to examine how weight control may vary among demographic groups.

**Methods:**

Data were collected as part of a national health and wellbeing survey of secondary school students in New Zealand (n = 9,107) in 2007. Item response theory analyses were conducted to determine the underlying constructs of weight control behaviors and the behaviors that discriminate unhealthy from healthy weight control.

**Results:**

The current study confirms that there are two underlying constructs of weight loss behaviors which can be described as healthy and unhealthy weight control. Unhealthy weight control was positively correlated with depressive mood. Fasting and skipping meals for weight loss had the lowest item thresholds on the unhealthy weight control continuum, indicating that they act as ‘red flags’ and warrant further discussion in routine clinical assessments.

**Conclusions:**

Routine assessments of weight control strategies by clinicians are warranted, particularly for screening for meal skipping and fasting for weight loss as these behaviors appear to ‘flag’ behaviors that are associated with poor mental wellbeing.

## Background

Weight control behaviors are common among adolescents. Data from the 2009 Youth Risk Behavior Survey suggest that more than 60% of adolescents exercised for weight loss in the 30 days before the survey and 4% vomited, 5% took diet pills, and 10% went without eating [1]. In a nationally representative sample of adolescents in New Zealand, approximately two-thirds of females and one-third of males had attempted weight loss in the previous year with concerning proportions of young people fasting, vomiting, or smoking more cigarettes for weight loss [2]. Given the increasing prevalence of overweight and obese children and young people, weight control behaviors may be indicated for some obese/overweight adolescents as long as they are healthy weight control strategies (e.g. eating less junk food).

Unfortunately unhealthy weight control behaviors are common, especially among overweight young people, and there is abundant evidence that unhealthy weight loss behaviors lead to poor outcomes for adolescents. For example, adolescents who used diet pills, vomited, took laxatives, took diuretics, fasted, used food substitutes, skipped meals or smoked cigarettes for weight loss had high levels of depression and were more likely to develop suicidal behaviors into young adulthood [3]. Likewise, other studies have found that adolescents who fasted, used food substitutes, skipped meals, smoked cigarettes, took diet pills, vomited, took laxatives or used diuretics had poorer nutrient intakes [4] and adolescents who used more extreme weight control behaviors (defined as vomiting and/or taking diet pills) ate fewer fruits and vegetables and ate more high fat foods [5] than those who were not dieting or using only moderate weight control behaviors (defined as anything other than vomiting or diet pills). Furthermore, it has been reported that approximately 20% of severe dieters (defined as the top third of students reporting calorie counting, reducing food, and skipping meal strategies) go on to develop a new eating disorder [6]. In general, adolescents who diet (change how they eat in order to lose weight) are more likely to develop binge eating behaviors and gain more weight [7] over time, compared to those who do not.

Given the pervasiveness of weight control behaviors among young people and the potential for adverse outcomes, it is concerning that clinicians rarely ask young people about these behaviors or conduct routine screening of adolescents about weight control behaviors [8]. This may be due to uncertainty about which questions to ask, specifically around what weight loss behaviors are more likely to lead to adverse outcomes and whether certain weight loss strategies are healthy or unhealthy (e.g. skipping meals). It also may reflect a lack of awareness that overweight and obese young people are more likely to use unhealthy weight control strategies than their normal weight peers [9,10].

One of the challenges that health care professionals face is limited time to ask the most important questions on all aspects of a young person’s life. The standard protocol for interviewing adolescents in a healthcare setting includes discussion on numerous aspects of the lives of adolescents, including home, school, eating, friends, drugs, sexuality, depression, and safety [11]. While comprehensively assessing body satisfaction, perceived weight and eating and exercise behaviors systematically may give an indication of a young person’s risk, clinicians may not always have the time to fully enquire about every possible of weight loss strategy. Therefore it would be useful for clinicians to better understand which weight loss behaviors are healthy or unhealthy, which unhealthy weight loss behaviors are more severe and require urgent attention, and which weight loss behaviors are less severe but signify ‘red flags’ as unhealthy weight control strategies.

Item response theory (IRT) analyses of weight control behaviors can provide clinicians with information on how various weight loss behaviors relate to an underlying continuum of risk, from less severe to more severe behaviors and therefore a better understanding of which behaviors place young people at higher risk of harm. IRT can be used to identify the validity of underlying latent constructs of behavior (e.g. unhealthy weight control, healthier weight control) by relating these continuums to other outcomes such as mental health concerns. IRT analyses produce two main parameters of interest to clinicians: item thresholds and factor loadings. The item thresholds parameters correspond to item difficulty and reflect the endorse-ability of a behavior (e.g. vomiting) along the continuum of the latent construct (e.g. unhealthy weight control). Item factor loadings identify those behaviors which are more accurately able to discriminate students along the continuums of the latent constructs.

Item response theory analyses also provide information of possible systematic biases or measurement equivalence between demographic groups [12]. These arise when individuals with equivalent levels of underlying risk from unhealthy weight loss behaviors respond differently to specific weight loss behaviors depending on their demographic characteristics. For example, males and females may have differing levels of risk from endorsing the same weight loss behavior. IRT analyses that assess measurement equivalence between demographic groups are important for clinicians to better understand how weight control behaviors may differ in their meaning between demographic groups and to lessen the chance of unbiased diagnoses.

The specific objectives of the current study are: 1. to determine which weight loss behaviors are on the unhealthy and healthy continuums of weight control, 2. to identify weight loss behaviors that are ‘severe’ (or difficult) and may require urgent attention and behaviors that are less difficult, but are the ‘red flags’ signifying unhealthy weight loss strategies, 3. to explore the association of unhealthy and healthy weight control with symptoms of depression and wellbeing among students, and 4. to examine how weight control may vary among demographic groups and which demographic groups are engaging with healthy and unhealthy weight control behaviors.

## Methods

Data for the current study were collected as part of Youth’07, a national survey of the health and well-being of New Zealand secondary school students (approximate ages 13-18). Full details of the methodology and survey design of the Youth’07 survey are described elsewhere [13] with a brief description outlined below. Data for the Youth’07 survey were collected in 2007.

Participating students were randomly selected through a two-stage clustered sampling design. First, 115 schools were randomly selected for participation then students were randomly selected for participation from the school roll. In total, 9,107 students agreed to participate in the survey. The final response rate for schools was 84% and for students 74%. Among the most common reasons for students not participating were being absent from school or being unavailable on the day of the survey [13].

School principals consented to participation in the survey on behalf of the Boards of Trustees. Selected students and their parents were provided with information sheets about the survey. Students consented themselves to participate in the study on the day of the survey. The University of Auckland Human Subject Ethics Committee granted ethical approval for the study.

All data were collected during the school day. On the day of the survey, students arrived at a designated room where they were given an anonymous login code to access the survey. The Youth’07 survey included a 622 item multimedia questionnaire administered on an internet tablet, anthropometric measurements, and identification of their census mesh block number (based on their residential address) to determine small-area neighborhood deprivation.

### Measures

*Weight lossattempt* was assessed with the question, “In the last 12 months, have you ever tried to lose weight (yes/no)?” Students who answered yes were then asked about their *weight lossstrategies* with the question, “During the past year, have you done any of the following things to lose weight or stop gaining weight?” Students could select as many strategies as appropriate from a list including I exercised, I ate less fatty food, I ate fewer carbohydrates, I ate a high protein diet (eggs, meat, etc), I counted calories, I ate fewer sweets and less sugar, I fasted or did not eat for more than one day, I skipped one or more meals a day, I made myself vomit, I took diet pills or other pills, I smoked cigarettes. *Age*, *gender* and *ethnicity* were determined by self-report.

*Wellbeing* was assessed with the WHO-Five Wellbeing Index [14] which measures three underlying constructs of positive mood, vitality, and general interests. The index includes five items rated on a six point Likert scale from 0 (at no time) to 5 (all of the time). The responses were summed to derive an overall score with higher scores indicating better wellbeing (Cronbach’s alpha = 0.89). Depression was measured using the Reynolds Adolescent Depression Scale – short form [15] which has acceptable reliability and validity for New Zealand adolescents (Cronbach’s alpha = 0.88) [16].

### Analysis

Given the branching nature of the questionnaire and research topic of interest, our analyses includes only those students (50.1%; n = 4,358) who have made a weight loss attempt in the past year. Students who have made weight loss attempts in the past year are more likely to be female (67%) than male (36%), but there were no differences by age.

A confirmatory factor analysis and exploratory SEM models with categorical indicators were used to confirm the hypothesized factor structure of the healthy and unhealthy weight control behaviors and derive the IRT parameters [17,18]. Overall fit of these models was assessed by goodness-of-fit indices that are sample size independent using criteria suggested by Hu and Bentler [19]: the comparative fit index (CFI) greater than 0.95, Tucker-Lewis fit index (TLI) greater than 0.95, root mean square error of approximation (RMSEA) less than .06.

Item characteristic curves are used to depict the relationships between the weight loss behaviors and the latent weight loss dimensions underlying each behavior and are characterized by item difficulty and item discrimination parameters. Item difficulty indicates the position of the item characteristic curve in relation to the underlying latent continuum and item discrimination measures the accuracy in which each item distinguishes between participants with levels of the latent dimension above versus those with levels below the item’s difficulty. To assess the concurrent validity of the underlying latent dimensions of weight loss behaviors, correlation of the latent factors with mental health measures were performed.

Finally, MIMIC modeling was used to examine the presence of differential criterion functioning by demographic variables and the association between the latent weight control factors and demographic variables [20]. Factor loadings and modification indices were examined after the addition of demographic variables to the model. Modification indices give the expected drop in chi-square if the parameter in question is allowed to be freely estimated. Direct effects between demographic variables and indicator variables were then examined for parameters with modification indices over 10. The presence of differential criterion functioning indicates measurement non-equivalence or item response bias across groups. All analyses took into account the non-independence of observations from students within the same school and unequal probability of selection. Analyses were conducted using the Mplus statistical software package [21].

## Results

### Underlying constructs of weight control behaviors

Among students who had attempted weight loss in the past year, exercising (90%), eating less fatty foods (72%), and eating fewer sweets (52%) were the most commonly used strategies for weight loss (Table 1). To confirm the hypothesized dimensions of health and unhealthy weight control strategies, a confirmatory factor analysis (CFA) model was fitted to the data. Healthy weight control strategies were hypothesized to include: exercising, eating less fatty food, counting calories, eating fewer sweets/less sugar, eating fewer carbohydrates, and eating high protein diet. Vomiting, using diet pills, smoking cigarettes, fasting and skipping meals were hypothesized as unhealthy weight control strategies. This model did not fit the data very well (*χ*^2^ = 312.88, df = 44, p < 0.001, RMSEA = 0.053, CFI = .86, TLI = .83). Thus, an exploratory ESEM model was fitted to the data using geomin rotation. This model fitted the data reasonably well (*χ*^2^ = 152.56, df = 34, p < 0.001, RMSEA = 0.04, CFI = .94, TLI = .90). The factor loadings are shown in Table 1. For the latent healthy factor, all the expected healthy weight loss strategies loaded on to this latent factor and vomiting and smoking calories had significant negative loadings on this factor. For the latent unhealthy weight loss factor, all the expected unhealthy weight loss behaviors loaded significantly on to this factor and exercising had a significant negative loading. Of interest, counting calories and eating fewer carbohydrates had moderate loadings on this factor as well.

**Table 1:** Factor loadings for healthy and unhealthy latent factors

		**Factor loadings**			
		**Healthy**		**Unhealthy**	
	**Prevalence (%)**	**Estimate**	**S.E.**	**Estimate**	**S.E.**
Vomited	7.8	**-0.111**	**0.038**	**0.778**	**0.022**
Diet pills	3.5	0.042	0.056	**0.661**	**0.041**
Smoked cigarettes	9	**-0.232**	**0.038**	**0.527**	**0.034**
Fasted	12.5	0.004	0.015	**0.814**	**0.021**
Skipped meals	31.4	0.004	0.018	**0.775**	**0.021**
Exercised	89.8	**0.484**	**0.029**	**-0.238**	**0.035**
Ate less fatty food	71.5	**0.888**	**0.027**	-0.019	0.02
Counted calories	7.7	**0.422**	**0.03**	**0.319**	**0.041**
Ate fewer sweets/less sugar	52.3	**0.709**	**0.022**	0.01	0.011
Ate fewer carbohydrates	18.5	**0.571**	**0.024**	**0.26**	**0.036**
Ate high protein diet	14.8	**0.508**	**0.028**	0.078	0.031

Bold indicates significant p < 0.01.

### Identifying the ‘red flags’ for unhealthy weight control

**Figure 1:**
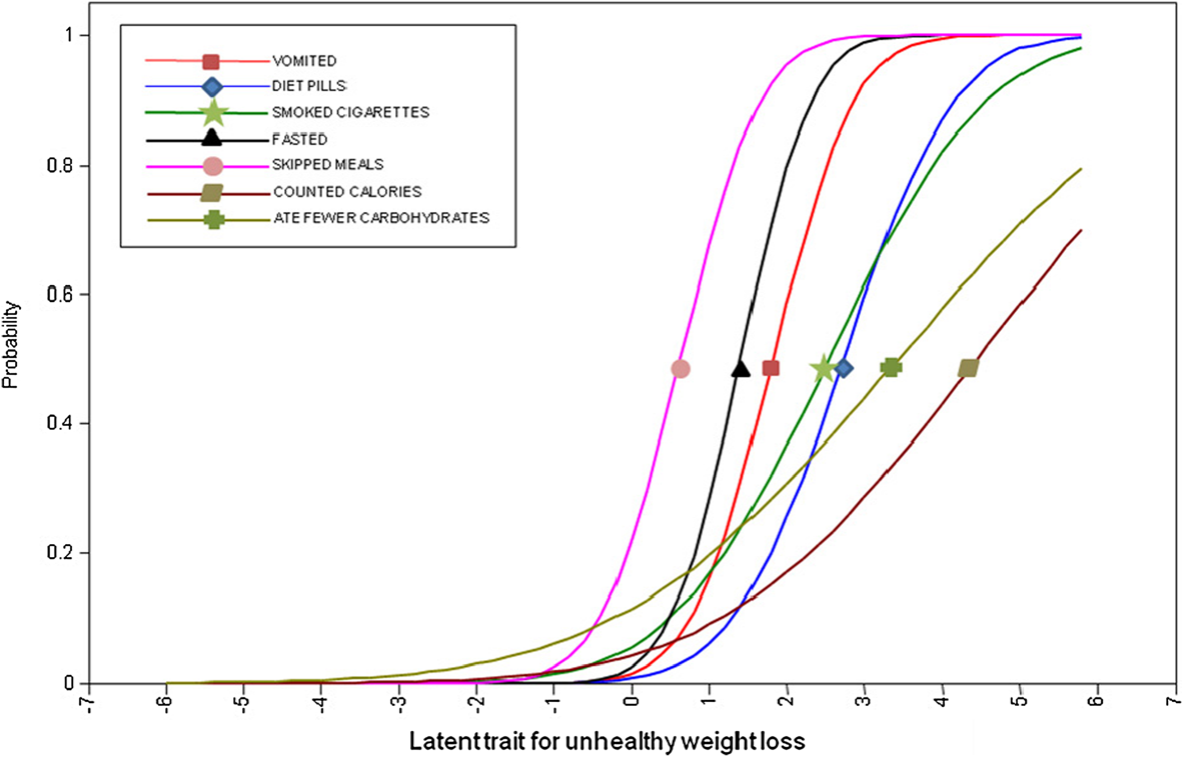
Item characteristic curves for unhealthy weight control.

**Figure 2:**
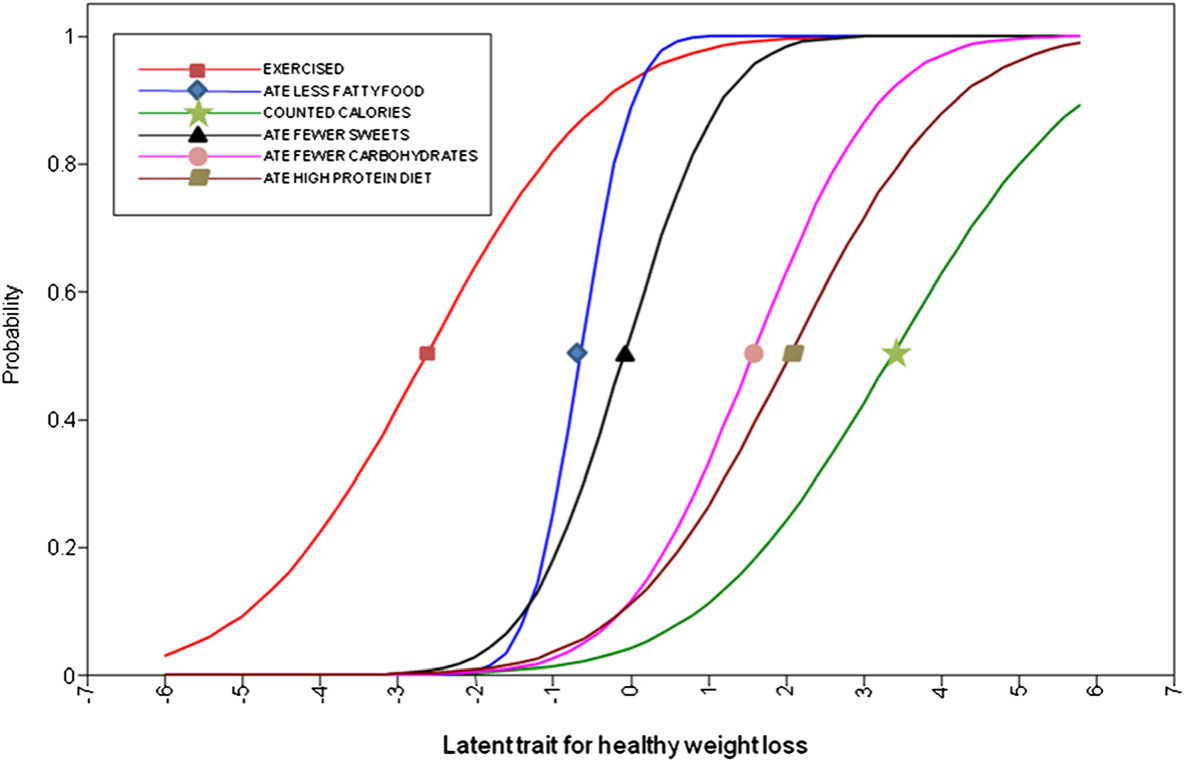
Item characteristic curves for healthy weight control.

Table 2 shows the item response parameters and Figure 1 and 2 display the item characteristic curves for the healthy and unhealthy weight loss behaviors that loaded significantly (p < 0.05) and positively on the two respective latent factors. Along the latent healthy weight loss continuum, exercising had the lowest item difficulty suggesting that this behavior was the easiest healthy weight loss behavior for students to engage in. In comparison, counting calories had the highest item difficulty among the healthy weight loss behaviors reflecting the complexity of this behavior. Counting calories and eating fewer carbohydrates also had the highest item difficult along the unhealthy weight loss continuum. Skipping meals and fasting had the lowest item difficulty along the unhealthy weight control continuum indicating that these behaviors may the easiest unhealthy weight loss behaviors for students to engage in. This suggests that these behaviors may act as the ‘red flags’ and may be important screening questions for assessing unhealthy weight control.

**Table 2:** Item response theory parameters of unhealthy weight control

	**Healthy**		**Unhealthy**	
	**Difficulty**	**Discrimination**	**Difficulty**	**Discrimination**
Vomited	-	-	2.328	1.238
Diet pills	-	-	2.144	0.881
Smoked cigarettes	-	-	2.546	0.620
Fasted	-	-	1.412	1.401
Skipped meals	-	-	0.630	1.226
Exercised	-2.628	0.553	-	-
Ate less fatty food	-0.641	1.931	-	-
Counted calories	3.370	0.465	4.458	0.337
Ate fewer sweets/less sugar	-0.082	1.005	-	-
Ate fewer carbohydrates	1.569	0.696	3.446	0.269
Ate high protein diet	2.053	0.590	-	-

The weight loss items all exhibited reasonable discrimination (range 0.2 - 1.9) in distinguishing along the healthy and unhealthy weight control continuums, as indicated by steep lines in Figure 1 and 2. Counting calories and eating fewer carbohydrates had the lowest item discriminations among the unhealthy weight loss behaviors; this may reflect that these behaviors also loaded onto the latent healthy weight control factor. It is notable that exercising had the lowest item discrimination along the healthy weight loss continuum; this likely reflects the many reasons that students engage in exercise other than weight control.

### Relationship between weight loss behaviors and mental wellbeing

**Figure 3:**
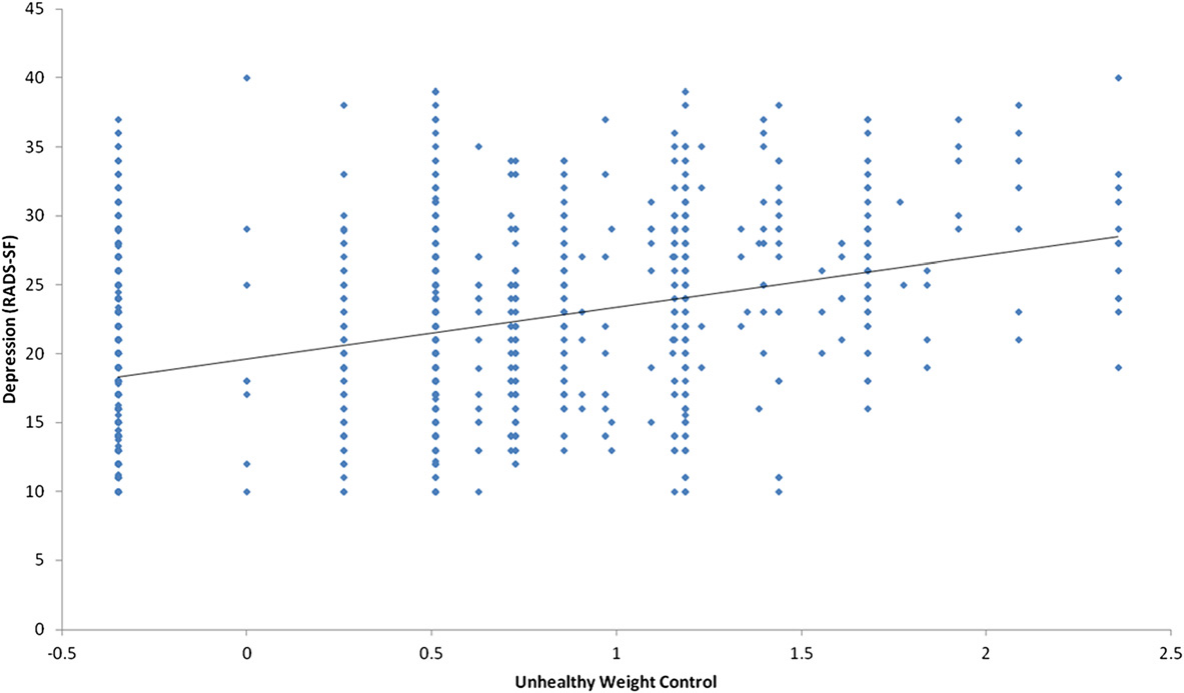
Scatterplot of relationship between unhealthy weight control and depression.

**Figure 4:**
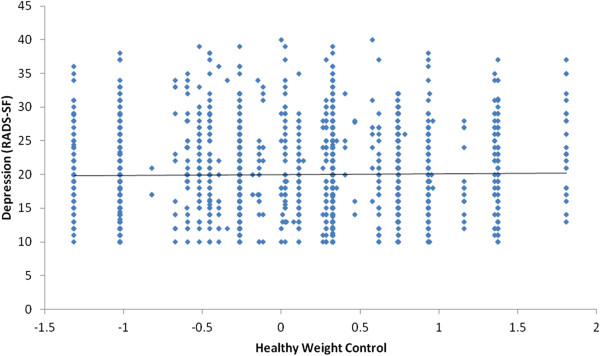
Scatterplot of relationship between healthy weight control and depression.

Students who endorsed more difficult unhealthy weight loss behaviors had significantly lower wellbeing scores (*r* = -0.36, p < 0.001) and significantly higher depression scores (*r* = 0.48, p < 0.001) than students who reported ‘easier’ unhealthy weight control behaviors (Figure 3). There was a minimal relationship between students using more difficult healthy weight loss behaviors and higher wellbeing scores (*r* = 0.08, p < 0.001) and lower depression scores (*r* = -0.05, p < 0.001) compared with students reporting ‘easier’ healthy weight loss behaviors (Figure 4).

### Does weight control vary by demographic groups?

Fitting a MIMIC model by including the covariate variables age, sex and ethnicity reduced the model fit indices (*χ*^2^ = 602.86, df = 79, p < 0.001, RMSEA = 0.039, CFI = .88, TLI = .83). Results of differential criterion functioning analyses showed several weight control behaviors had significant direct loadings on the age, gender and ethnicity variables. This suggests that the thresholds of the weight loss items in relation to the underlying weight control constructs may vary across these demographic groups. For example, males had a higher probability of endorsing eating a high protein diet and fasting to lose weight than female students for given level of the latent weight control factors. In contrast, female students were more likely to report vomiting to lose weight than male students for a given level of the latent weight control factors. Thirteen direct effects were added to the overall model with an improvement in model fit (*χ*^2^ = 324.57, df = 66, p < 0.001, RMSEA = 0.030, CFI = .94, TLI = .90). There was no substantial change to the factor loadings following these modifications and the regression of the covariates on the latent healthy and unhealthy weight control factors did not change substantially.

The latent mean score for the healthy weight loss factor was 0.14. We found that males were significantly more likely than females to have a lower latent healthy weight loss factor scores (mean latent score = -0.09, p < 0.001). Compared to NZ European students, Maori students and Pacific students had significantly lower latent healthy weight loss factor scores (mean latent score = -0.23, p < 0.001 and -0.23, p < 0.001, respectively). The latent mean scores for the unhealthy weight loss factor was -0.06. Females were significantly more likely than males to have a higher latent unhealthy weight loss scores (mean latent score = 0.5, p < 0.001). Students of Maori or Pacific ethnicities had significantly higher latent unhealthy factor scores (mean latent score 0.64, p = 0.02 and 0.69, p = 0.004, respectively). Asian ethnicity was associated with lower levels of unhealthy weight control (mean latent score = 0.35, p < 0.001). Taken together, these findings suggest that females were more likely to endorse more difficult weight control behaviors on both the healthy and unhealthy weight control constructs. Moreover, Maori students and Pacific students were less likely to endorse more difficult weight control behaviors on the healthy weight control construct, but more likely to endorse the more difficult weight control behaviors on the unhealthy weight control construct.

## Discussion

Drawing on a large, nationally representative population of adolescents, the current study has been able to confirm that there are two underlying constructs of weight control behaviors which are consistent with what previous literature has referred to as healthy and unhealthy weight control [4,5,9,10,22,23]. The unhealthy construct in the current analyses included behaviors such as vomiting, using diet pills, smoking cigarettes, fasting and skipping meals. The current study also demonstrated that unhealthy weight control is positively associated with depression. This is consistent with previous research which has suggested that using unhealthy weight control behaviors is associated with poor outcomes in terms of their nutritional status and emotional wellbeing [3,4,24].

We found that skipping meals and fasting were among the most common unhealthy weight loss behaviors and had the lowest item difficulty on the unhealthy weight control continuum. This indicates that these behaviors may warrant discussion in routine clinical assessments and may be appropriate to use as screening questions for unhealthy weight control behaviors. In the current sample, skipping meals was a commonly used weight loss strategy with 15% of all students and 30% of students who have attempted weight loss in the past year reporting the behavior [2]. Skipping meals is a commonly reported weight loss strategy among American adolescents as well, with previous studies estimating that 20-30% of adolescents in the general population report this behavior [25,26]. Furthermore, skipping meals is not an effective weight loss strategy as available research on successful weight loss suggests that regular meal consumption, particularly breakfast, is important for weight loss maintenance. [27,28] Thus, regular screening for fasting and skipping meals specifically, may be as quick and useful marker for other risk behaviors, including more unhealthy weight control.

In the current study, among the more difficult indicators of the unhealthy weight control construct were use of diet pills, smoking for weight loss and vomiting. Some previous research has referred to these types of weight control behaviors as extreme weight control behaviors and demonstrated that young people who use these weight control behaviors are at risk for multiple poor outcomes. For example, Story et al. [24] found that young people who had vomited or used diet pills for weight loss in the past week were less likely to eat fruits and vegetables and more likely to consume high fat foods. Similarly, Neumark-Sztainer et al. [29] demonstrated that young people who had vomited or used diet pills for weight loss were more likely to engage in a number of risk-taking behaviors, such as substance use and unprotected sexual activity. More recently, Crow et al. [3] observed that young female adolescents who used diet pills, laxatives, diuretics or vomited for weight loss were significantly more likely to report suicidal attempts and suicidal ideation into young adulthood than those who did not.

It was of interest that two behaviors, counting calories and eating fewer carbohydrates, loaded positively onto both the healthy and unhealthy weight control constructs and that these were more difficult behaviors on both constructs. Thus, when young people report the use of these behaviors for weight control in clinical settings, clinicians need to discuss the range of other weight control behaviors students are engaging in to be able to determine how healthy their weight loss strategies are.

The healthy weight control construct identified in the current study is consistent with recognized weight control recommendations encouraging dietary improvements and exercise, [30] such as eating more vegetables and fewer sweets. Yet there is mixed evidence to suggest that adolescents who engage in weight control behaviors commonly described as healthy weight control achieve better health and wellbeing [7,23-25,27]. It is of interest, then, that we observed only a slight relationship between healthy weight control behaviors and positive emotional wellbeing. As discussed above, the bulk of the previous research has looked at the relationship between emotional wellbeing and unhealthy weight control behaviors rather than healthy weight control behaviors.

In the current study, we found evidence of differential item functioning suggesting measurement non-equivalence or item response bias across demographic groups. It is known that the prevalence of weight loss behaviors varies by age, gender and ethnicity among New Zealand young people [2], and internationally as well [31]. The varying prevalence of these behaviors by demographic groups and differential item functioning found in the current study suggests that there may be differing underlying cultural and gendered meanings of these behaviors and weight control more generally. One of the limitations of the current study is that our analysis of differential item functioning only concerns non-invariance of threshold parameters and not factor loadings across groups. While we were able to model some of the more significant direct effects to account for non-invariance of item threshold across groups, it is likely that non-invariance of factor loadings between groups also exists. However after the addition of direct effects the relationship between covariates and the healthy and unhealthy latent factors did not change substantially.

While the comparison of latent factor mean values between groups relies on the assumption of measurement invariance, our results indicated that young people who were female or of Maori or Pacific ethnicities were more likely to endorse more difficult weight control behaviors on the unhealthy weight control construct. Thus, these young people who report weight control behaviors may warrant extra clinical attention and monitoring to prevent poor outcomes associated with unhealthy weight control.

The strengths of the current study lie in its large, nationally representative sample of young people, well validated measures of both depression and positive mood, and use of analytic techniques to confirm what previous researchers have hypothesized. That said, there are a few limitations to the current study that warrant consideration in its interpretation. First, while our study is generalizable to secondary school students in New Zealand, this may not be true for young people in other parts of the world. This is particularly true for the weight control behaviors included in the current study as they may not be inclusive of the most commonly employed weight control behaviors used by young people in other regions of the world. For example, our study did not include items on diuretics or laxatives, yet these behaviors are reported by approximately 2% of young people in the US. [32] The current study is also limited in its depth of questioning around the weight control behaviors. For example, more information on frequency of weight control behaviors and the effects of the unhealthy weight control strategies would add depth to our understanding of the issue, but was not easily addressed in this large, broad health survey.

## Conclusions

Weight control behaviors are common among young people and unhealthy weight control behaviors pose unique risks to their wellbeing. Routine assessment of weight control strategies by clinicians are warranted, particularly for screening for meal skipping and fasting for weight loss as these behaviors appear to ‘flag’ strategies that are associated with poor mental wellbeing.

## Competing interests

All authors declare that they have no competing interests to disclose.

## Author’s contributions

JU conceived the research questions, interpreted the data, and drafted the manuscript. SD and ER acquired the data, conducted and oversaw data analyses, and revised the manuscript for intellectual content. SA and SC acquired the data and revised the manuscript for intellectual content. All authors read and approved the final version of the manuscript.
